# Refractory episodic vertigo: role of intratympanic gentamicin and vestibular evoked myogenic potentials^☆^^☆☆^

**DOI:** 10.1016/j.bjorl.2015.11.019

**Published:** 2016-03-28

**Authors:** Erika Celis-Aguilar, Ramon Hinojosa-González, Olivia Vales-Hidalgo, Heloisa Coutinho-Toledo

**Affiliations:** a Universidad Autónoma de Sinaloa, Centro de Investigación y Docencia en Ciencias de la Salud (CIDOCS), Departamento de Otorrinolaringología, Culiacán, Sinaloa, Mexico; b Instituto Nacional de Neurología y Neurocirugía Manuel Velasco Suárez, Department of Neurotology, Ciudad de México, Mexico; c Hospital Médica Sur, Ciudad de México, Mexico

**Keywords:** Vestibular evoked myogenic potentials, Refractory vertigo, Intratympanic gentamicin, Meniere disease, Potencial evocado miogênico vestibular, Vertigem refratária, Gentamicina intratimpânica, Doença de Ménière

## Abstract

**Introduction:**

Even today, the treatment of intractable vertigo remains a challenge. Vestibular ablation with intratympanic gentamicin stands as a good alternative in the management of refractory vertigo patients.

**Objective:**

To control intractable vertigo through complete saccular and horizontal canal vestibular ablation with intratympanic gentamicin treatment.

**Methods:**

Patients with refractory episodic vertigo were included. The inclusion criteria were: unilateral ear disease, moderate to profound sensorineural hearing loss, and failure to other treatments. Included patients underwent 0.5–0.8 mL of gentamicin intratympanic application at a 30 mg/mL concentration. Vestibular ablation was confirmed by the absence of response on cervical vestibular evoked myogenic potentials and no response on caloric tests. Audiometry, electronystagmography with iced water, and vestibular evoked myogenic potentials were performed in all patients.

**Results:**

Ten patients were included; nine patients with Meniere's disease and one patient with (late onset) delayed hydrops. Nine patients showed an absent response on vestibular evoked myogenic potentials and no response on caloric tests. The only patient with low amplitude on cervical vestibular evoked myogenic potentials had vertigo recurrence. Vertigo control was achieved in 90% of the patients. One patient developed hearing loss >30 dB.

**Conclusions:**

Cervical vestibular evoked myogenic potentials confirmed vestibular ablation in patients treated with intratympanic gentamicin. High-grade vertigo control was due to complete saccular and horizontal canal ablation (no response to iced water in electronystagmography and no response on cervical vestibular evoked myogenic potentials).

## Resumo

**Introdução:**

Ainda hoje, o controle da vertigem intratável permanece um desafio. A ablação vestibular com gentamicina intratimpânica permanece como uma boa alternativa no tratamento de pacientes com vertigem refratária.

**Objetivo:**

Controlar a vertigem intratável por meio de ablação vestibular completa dos canais sacular e horizontal com gentamicina intratimpânica como tratamento.

**Método:**

Pacientes com vertigem refratária episódica foram incluídos. Os critérios de inclusão foram doença unilateral da orelha, perda auditiva neurossensorial de moderada a profunda e fracasso com outros tratamentos. Os pacientes incluídos receberam uma aplicação de 0,5–0,8 mL de gentamicina intratimpânica com concentração de 30 mg/mL. A ablação vestibular foi confirmada pela ausência de resposta no teste de potencial evocado miogênico vestibular cervical (PEMVc) e nenhuma resposta nas provas calóricas. Audiometria, eletronistagmografia com água gelada e potencial evocado miogênico vestibular foram realizados em todos os pacientes.

**Resultados:**

Ao todo, dez pacientes foram incluídos: nove com doença de Ménière e um com hidropisia tardia. Nove pacientes apresentaram ausência de resposta no teste de potencial evocado miogênico vestibular e nenhuma resposta na prova calórica. O único paciente com baixa amplitude no PEMVc apresentou recorrência da vertigem. O controle da vertigem foi obtido em 90% dos pacientes. Um paciente desenvolveu perda auditiva > 30 dB.

**Conclusões:**

O PEMVc confirmou ablação vestibular nos pacientes tratados com gentamicina intratimpânica. O alto grau de controle da vertigem foi devido à ablação completa do sáculo e canal horizontal (sem resposta à água gelada na eletronistagmografia e ausência de resposta no PEMVc).

## Introduction

Vertigo control is the main outcome in the treatment of Meniere's disease; nevertheless, many patients do not respond to conservative measures. More invasive procedures are preserved for those patients with intractable vertigo and no response to medical treatment. Surgical treatments, particularly vestibular nerve section, have a high success rate. Nonetheless, as a surgical procedure, side effects such as headache, bleeding, cerebrospinal fluid (CSF) leak, or facial paralysis, among others, are possible. For this reason, intratympanic gentamycin treatment is gradually replacing these surgical procedures.

In 1957 Schuknecht^1^, ^2^ was the first to describe the use of aminoglucosides in the treatment of Meniere's disease. He described the instillation of streptomycin in the middle ear of five patients, with good vertigo control, although with adverse cochlear side effects, as profound hearing loss developed in all patients.

Since then, numerous studies of intratympanic gentamicin have demonstrated a 76–96% vertigo control rate, with a risk of hearing loss around 20–30%.^1^, ^2^

Interestingly, gentamicin instillations are currently still controversial. Dose, number of applications, and period between each application has not reach a consensus between otologists. In 2004, a meta-analysis^2^ described five different techniques of gentamicin applications: daily (three daily applications for at least four days), weekly dose (one each week for a total of four doses), low dose (one or two applications, with new treatment if vertigo recurrence), continuous application with microcatheter, and titration therapy (weekly or daily dose until vestibular or cochlear symptoms occur). Chia et al.^2^ concluded, according to this meta-analysis, that the best method of gentamicin application was titration, with vertigo control as high as 81.7%. These authors did not find statistical significance difference between partial *vs.* complete vestibular ablation.

On the other hand, cervical vestibular evoked myogenic potentials (cVEMPs) were first described by Colebatch in 1994^3^; since then, cVEMPs have been a known test for saccular function and inferior vestibular nerve. In humans, intense auditory clicks and tone bursts stimulate afferent saccular pathways that inhibit sternocleidomastoid muscle tone, which is recorded on this test. The result is a positive wave p1, followed by a negative wave n1.

Furthermore, saccular function has been found altered in patient with recently diagnosis of Meniere's disease.^4^ It is believed that complete vestibular function, including saccular function, should be measured in gentamicin treatment. Moreover, the rehabilitation of a patient who undergoes gentamicin treatment depends on the amount of vestibular damage present.^5^

Disadvantages of intratympanic gentamicin are mainly hearing loss^6^ and chronic subjective dizziness.

Evaluation of the saccular function is paramount if the goal of vestibular ablation with intratympanic gentamicin is complete ablation, and cVEMPs could add to the diagnostic work-up and follow-up of these patients. This test could also show vestibular residual function in a patient with recurrent vertigo post-gentamicin treatment.

Complete vestibular ablation *vs*. partial ablation is a subject of controversy. Unfortunately, reports on how this vestibular ablation is obtained are varied. To determine complete vestibular ablation, studies should include evaluation of semicircular canals, as well as utricular and saccular function.

Vestibular ablation in this study was defined as zero response on the caloric tests with ice water and absent response on cVEMPs, *i.e.*, complete ablation of saccular and horizontal semicircular canal function.

The objective of this study was to assess vertigo control in patients with intractable refractory vertigo treated with intratympanic gentamicin and vestibular ablation, confirmed by an absent response on cVEMPs and zero response in the ice water test on electronystagmography (ENG).

## Methods

### Study design

Retrospective, clinical chart review.

### Setting

Tertiary care center.

### Patients

This study included patients with intractable recurrent vertigo, unilateral vestibular disease, and complete vestibular tests, from January 2002 to December 2010. Weekly gentamicin application was conducted in all patients. The application consisted of 0.5 ml to 0.8 ml of gentamicin, with a concentration of 30 mg/mL. This solution was buffered with bicarbonate. Inclusion criteria: All patients fulfilled the criteria for Meniere's disease according to the 1995 AAO-HNS committee on hearing and equilibrium, had age >18 years, moderate to profound hearing loss, and failure to other medical treatments including dexamethasone intratympanic application.

### Measurements

All patients underwent pure tone audiometry and speech audiometry with an Interacoustic AD29 audiometer. ENG was performed with micromedical equipment. Oculomotor tests (saccades, gaze testing, optokinetic, smooth pursuit tracking), positional tests, and caloric tests were performed in all patients. Vestibular asymmetry was defined through the caloric tests, with an asymmetry of 30% compared to the other ear. Zero response was defined as absence of nystagmus with iced water.

cVEMPs were performed with Nicolet Viking Select consisted of electrodes placed on the sternocleidomastoid, sternum, and ipsilateral mastoid (ground electrode). Sternocleidomastoid tone was obtained by effort elicited by the patient by means of a pole system with a weight of 2 kg and a headband. The patient thrusts forward his/her forehead, maintaining muscle tone. Auditory stimuli consisted of clicks, three per second at 105 dB (with a contralateral white noise of 45 dB, and a filter of 10 Hz–1.5 kHz). Recordings were conducted for 100 ms (milliseconds).

Informed consent was obtained from all patients. This study was approved by the institutional ethics committee.

### Intervention

Protocol of intratympanic gentamicin application: patient lays flat with 45° of head rotation, tetracaine solution is applied to ear canal, under microscopic view the anesthetic is suctioned, and with a 1 mL syringe with 30 mg gentamicin, buffered with 1/3 NaHCO_3_; 0.4 ml to 0.6 ml is applied to the middle ear, in the inferior quadrants. The patients rests in that position for over one hour, and is instructed to not speak or swallow. Weekly applications were performed until there was an absent response both to ice water ENG and cVEMPs.

### Follow-up

Patients were followed up with evaluations at 30 days, and three, six, and 12 months. Posterior to these evaluations, follow up was done annually. All patients underwent a vestibular rehabilitation program, which consisted of enhancing the vestibulo-ocular reflex, sensory substitution, and postural control; each protocol was individualized. During the patients’ subsequent visits, audiometry was performed, as well as ENG with ice water and measurement of the functional scale of Meniere's disease.

### Statistical analysis

Statistical analysis was performed using SPSS 15.0. The statistical analysis included descriptive statistics (mean and standard deviation).

## Results

Ten patients were included with complete vestibular tests, both ENG and cVEMPs. Nine patients had Meniere's disease and one patient had delayed endolymphatic hydrops.

All patients underwent intratympanic gentamicin application. The mean number of applications was 4.3 (range 1–7). Follow-up was from one to seven years, with a mean of 4.25 years.

Vertigo control was 90%. One patient developed hearing loss >30 dB. See Table 1 for results.

**Table 1:** Patients’ clinical characteristics, intervention, and follow up.

No.	Age	Sex	Caloric test (°)	Caloric test (°)	cVEMPs Post	No. IT	Vertigo	Follow up (years)	PTA dB difference
			Before	After					
1	69	F	20	0	Low amplitude	5/11	+	3	−2.5
2	55	M		0	NR	7	–	1	11.25
3	25	M	0	0	NR	1	–	4	12.5
4	42	F	6	0	NR	6	–	3	−1.67
5	60	F	53	0	NR	5	–	4	26.25
6	46	F	12	0	NR	3	–	5	−8.75
7	38	F	5 iced water	0	NR	2	–	7	−2.5
8	45	M		0	NR	4	–	1.5	−41.6
9	64	F	6	0	NR	5	–	7	9
10	33	M	8	0	NR	5	–	7	0

PTA difference, pure tone average difference (difference of dB in audiometry pre- and post-intervention); negative values represent hearing loss and positive values hearing gain in dB.

No. IT, number of intratympanic gentamicin applications; NR, no response; cVEMPs, cervical vestibular evoked myogenic potentials.

After gentamicin treatment, nine patients had absent response on cVEMPs and only one patient had persistent cVEMPs response. The latter patient (patient No. 1 on the table) had low amplitude cVEMPs with symptomatic recurrent vertigo. This patient required two sets of gentamicin applications; five and 11 applications, respectively.

All patients had zero response on ice water ENG posterior to gentamicin application. ENG oculomotor and positional tests were negative in all patients.

Two patients (patients No. 2 and 8) had ENG with vestibular asymmetry pre-treatment (>25%), although no grades of response were documented; therefore, this data is not included on Table 1.

### Previous treatment

All patients had no response to medical treatment (diet, diuretics, steroids, vasodilators, calcium antagonists). Interestingly, two patients had dexamethasone intratympanic application without good results, as well as one patient with endolymphatic sac decompression and one patient with possible incomplete vestibular nerve section.

### cVEMPS previous to gentamicin application

Even though all patients had documented vestibular paresis (horizontal semicircular canal paresis) previous to gentamicin treatment, only two (patients No. 3 and 4) had initial pre-treatment cVEMPs. Both patients had low amplitude response cVEMPs with posterior ablation of this response. These patients had Meniere's disease, with a mean age of 33.5 years.

### cVEMPs and vertigo control

Ninety percent of the patients had good vertigo control. The only patient with recurrent vertigo attacks was positive on cVEMPs posterior to the second course of gentamicin application. This patient rejected surgical treatment and was subsequently partially controlled with medical treatment.

### Hearing loss

Only one patient had hearing loss more than 30 dB. The rest of patients had a mean hearing loss of 5.70 dB.

### Functional class of Meniere's disease

Five patients had intermittent dizziness; they continue to work, drive, and engage in any activity. This corresponds to functional class levels 2 and 3.

### Technical difficulties of cVEMPs

Four patients had to be eliminated from the database because of bilateral absent response on cVEMPs. These technical difficulties were due to morphological neck variations (thick neck) or due to lack of cooperation from the patient (no sternocleidomastoid contraction).

## Discussion

The purpose of this study was to corroborate complete vestibular ablation by means of a zero response of ice water on ENG and an absent cVEMPs response. cVEMPs confirmed an absent saccular function and a non-functional posterior semicircular canal. On the other hand, the horizontal semicircular canal was measured by zero response on ice water with ENG. Thus, almost all of the functional vestibular epithelium was measured and complete vestibular ablation (saccular and horizontal semicircular canal function) could be achieved.

In this series, absent response on cVEMPs test after gentamicin treatment was present in all patients with good vertigo control.

Additionally, an abnormal response to cVEMPs could be an early symptom of Meniere's disease. In pre-symptomatic ears, it could be a sign of disease in the contralateral ear.^4^, ^7^

In the present study, two patients (patients No. 3 and 4) had positive cVEMPs pre-treatment, and interestingly, both had low amplitude response. This corroborates what has previously been described in the literature, that saccular damage is present in Meniere's disease.^4^, ^5^ Other authors have described an initial damage to semicircular canals with posterior saccular injury.^5^

Patient No. 3 was a controversial case, since there was zero response on ice water ENG but cVEMPs response was present; gentamicin treatment was chosen due to persistent saccular function. After gentamicin application, cVEMPs were negative and ice water ENG persisted negative after two years posterior to intervention. During this time posturography was normal, verifying complete vestibular rehabilitation. This patient is free of vertigo attacks.

cVEMPs could be of great diagnostic value when a patient presents with vertigo spells and no response on ice water ENG, since another vestibular epithelium, *e.g.* the saccule, could be responsible for the vertigo attacks.

Animal studies have proved that cervical VEMPs originate from type I cells in the saccule of guinea pigs.^8^ Vestibular cell regeneration after gentamicin has been described in both semicircular canals^8^ and the saccule.^7^ This vestibular regeneration is probably the cause of recurrent vertigo attacks post-gentamicin.^9^ However, other authors explain that this is due to the natural history of Meniere's disease, since the absence of vertigo attacks in some studies is due to an insufficient follow-up, giving a false idea of therapy success. Long term follow-up is strongly advised.^7^

In the present series, patient No. 1, after the first therapy of gentamicin, had grade 11 vestibular response on ice water ENG and also presented with low amplitude cVEMPs. After the second application of gentamicin, she persisted with vertigo attacks with positive cVEMPs, supporting the association of vestibular tests and symptoms, already described by numerous authors.^5^, ^7^, ^9^, ^10^

Helling et al.^5^ included in their study 19 patients with Meniere's disease. After the first gentamicin application cVEMPs were negative, they concluded that cVEMPs were not a reliable indicator of therapy success. This differs from the present results since the only patient with positive cVEMPS was the patient who had recurrent vertigo. Additionally, Picciotti et al.^11^ emphasized the used of cVEMPs to monitor therapy efficacy.

Presence of cVEMPs after intratympanic gentamicin could be an indicator of therapy failure, at least in the present study. More research is mandatory in order to confirm these results.

Additionally, intratympanic gentamicin delivery methods are highly variable. According to Chia et al.,^2^ the titration method demonstrated vertigo control of 81.7% *vs.* 75% for weekly delivery. The authors’ standard delivery method, as previously stated, is weekly applications until no response on ice water ENG. Comparing these results with those previously published, this study's rate of vertigo control could be superior to other methods of gentamicin delivery.

Complete vestibular ablation is still controversial, more so since the meta-analysis by Chia et al.^2^ did not confirm a statistical difference between partial and complete vestibular ablation (*p* = 0.179). The data show that complete vestibular ablation in this meta-analysis produced 92.1% vertigo control *vs.* 74.8% for partial ablation.

Other authors^5^ have preferred partial ablation, explaining that preserved canalicular function could achieve vertigo control, because it produced a more specific damage to the dark cells and thus provides endolymph production homeostasis. Nevertheless, the present study demonstrates that vestibular ablation (as measured by saccular and horizontal semicircular canal ablation) is effective for vertigo control.

Chia et al.^2^ described a 13.1% hearing loss with the weekly gentamicin delivery method. In the present series, only one patient had hearing loss greater than 30 dB, 10% of the study population. None of the patients had profound hearing loss secondary to the procedure, *vs.* 6.6% reported in other literature.^2^ The mean hearing loss was 5.7 dB. This rate of hearing loss is corroborated by other studies.^5^ Of relevance, there are other centers that apply intratympanic gentamicin in normal hearing subjects,^12^ emphasizing the low rate of expected hearing loss.

Interestingly, in this series, four patients had pure tone average (PTA) improvement. This could be due to the natural course of Meniere's disease, which includes fluctuating hearing loss.

Utricular function has been described to be preserved in 30%–40% of patients with intratympanic gentamicin treatment^5^; this differs from the saccule and semicircular canals, which are invariable injured by this treatment. Unfortunately, this study did not evaluate utricular function. Posturography has also been used in these patients. One study described an improvement in the vestibular component six months after gentamicin middle ear application. There are several venues of research that have been incompletely explored regarding this treatment that could be of use by future researchers.

The limitations of this study are its retrospective nature, the limited number of patients included, and the lack of pre-treatment cVEMPs in some patients. However, one strength of this study is the long-term follow-up, in some patients up to seven years.

Another limitation is the fact that the ENG caloric test represents only a low frequency test of vestibular function. The caloric test measures exclusively the horizontal semicircular canal function; therefore, it can be inferred that in this study, only partial vestibular ablation was measured. Nevertheless, the ENG also measures a dynamic range of other tests, such as positional testing and oculomotor tests (saccades, optokinetic, smooth pursuit, *etc.*), which were not altered in any of our patients.

The rotational chair test also evaluates the vestibulo-ocular reflex (VOR) and the horizontal semicircular canal; this test can be implemented for patients in whom it is difficult to perform caloric stimulation and adds accuracy to the ENG; nonetheless, this test was not performed in the present patients. Additionally, both the caloric test and the rotational chair test quantify the VOR reflex on the horizontal plane and at low-frequency. The vestibule ocular reflex can also be evaluated by the head impulse test, with or without Frenzel goggles; caloric tests could also be performed if necessary, with only Frenzel goggles at the emergency department.

Moreover, the authors are aware of the lack of other vestibular tests, such as ocular VEMPs, video head impulse test, and utricular function. In this study, although a complete absence of the saccule and horizontal semicircular canal function was achieved, complete vestibular function cannot be assumed because not all vestibular organs were measured.

Nevertheless, this study adds evidence on the use of intratympanic gentamicin for complete ablation of saccule and horizontal semicircular canal function. ENG caloric tests and cVEMPs should be use to corroborate vestibular ablation.

## Conclusions

cVEMPs confirmed vestibular ablation in patients treated with intratympanic gentamicin. High-grade vertigo control was due to complete saccular and horizontal semicircular canal ablation (no response to iced water in ENG and no response on cVEMPs).

## Conflicts of interest

The authors declare no conflicts of interest.

## References

[1] Lalwani A., McGuire J.F., Flint P., Haughey B., Lund V., Niparko J., Richardson M., Robbins K. (2010). Cummings otolaryngology – head and neck surgery.

[2] Chia S., Gamst A., Anderson J., Harris J. (2004). Intratympanic gentamicin therapy for Ménière disease: a meta-analysis. Otol Neurotol.

[3] Nguyen K., Welgampola M., Carey J. (2010). Test–retest reliability and age related characteristics of the ocular and cervical vestibular evoked myogenic potential tests. Otol Neurotol.

[4] Waele C., Tran Ba Huy P., Diard J.P., Freys G., Vidal P.P. (1999). Saccular dysfunction in Meniere's disease. Am J Otol.

[5] Helling K., Schönfeld U., Clarke A. (2007). Treatment of Meniere disease by low dose dosage intratympanic gentamicin application: effect on otolith function. Laryngoscope.

[6] Youssef T.F., Poe D.S. (1998). Intratympanic gentamicin injection for the treatment of Meniere's disease. Am J Otol.

[7] Ozluoglu L., Akkuzu G., Ozgirgin N., Tarhan E. (2008). Reliability of the vestibular evoked myogenic potential test in assessing intratympanic gentamicin therapy in Meniere's disease. Acta Otolaryngol.

[8] Lue J.H., Day A.S., Cheng P.W., Young Y.H. (2009). Vestibular evoked myogenic potentials are heavily dependent on type I hair cell activity of the saccular macula in guinea pigs. Audiol Neurotol.

[9] Waelw C., Meguenni R., Freyss G., Zamith F., Bellalimat N., Vidal P.P. (2002). Intratympanic gentamicin injections for Meniere's disease. Vestibular hair cell impairment and regeneration. Neurology.

[10] Welgampoala M., Colebatch J. (2005). Characteristics and clinical applications of vestibular evoked myogenic potentials. Neurology.

[11] Picciotti P.M., Fiorita A., Nardo W., Quaranta N., Paludetti G., Maurizi M. (2005). VEMPS and dynamic posturography after intratympanic gentamicin in Meniere disease. J Vestib Res.

[12] Silverstein H., Wazen J., Van Ess M., Daugherty J., Alameda Y.A. (2010). Intratympanic gentamicin treatment of patients with Ménière's disease with normal hearing. Otolaryngol Head Neck Surg.

